# On the limit value of compactness of some graph classes

**DOI:** 10.1371/journal.pone.0207536

**Published:** 2018-11-20

**Authors:** Tatiana Lokot, Alexander Mehler, Olga Abramov

**Affiliations:** 1 Retired, Faculty of Mathematics, Bielefeld University, Bielefeld, Germany; 2 Affiliation Faculty of Computer Science and Mathematics, Goethe-University Frankfurt, Frankfurt, Germany; 3 Affiliation CITEC/Faculty of Technology, Bielefeld University, Bielefeld, Germany; University of Tehran, ISLAMIC REPUBLIC OF IRAN

## Abstract

In this paper, we study the limit of compactness which is a graph index originally introduced for measuring structural characteristics of hypermedia. Applying compactness to large scale small-world graphs (Mehler, 2008) observed its limit behaviour to be equal 1. The striking question concerning this finding was whether this limit behaviour resulted from the specifics of small-world graphs or was simply an artefact. In this paper, we determine the necessary and sufficient conditions for any sequence of connected graphs resulting in a limit value of *C*_*B*_ = 1 which can be generalized with some consideration for the case of disconnected graph classes (Theorem 3). This result can be applied to many well-known classes of connected graphs. Here, we illustrate it by considering four examples. In fact, our proof-theoretical approach allows for quickly obtaining the limit value of compactness for many graph classes sparing computational costs.

## Introduction

Evidently, a hypertext forms a network of documents mostly linked on the basis of content-related connections. There is a range of studies applying the compactness measure itroduced in [2] in order to answer questions concerning the structure of hypermedia [1–8]. All these studies addressed compactness computing the values of particular graph invariants which implies high computational costs. In this paper, we take a different perspective considering the limit of compactness for different classes of connected graphs proof-theoretically. Our approach allows for omitting the computational step when the conditions below hold.

The paper is organized as follows. Section starts with repeating graph-theoretical notions used throughout the paper. Section outlines our main findings regarding the limit value of compactness. Section illustrates the application of our tool on four selected graph classes. Finally, Section summarizes our mathematical findings and gives an outlook on results obtained which are part of a subsequent publication. More specifically, in Section we give an overview of those graph classes for which compactness can be easily obtained applying our mathemaical tool (in fact, we have studied about 30 well-known graph classes, there are presumably more than those mentioned here for which our tool can be applied).

## Preliminaries

In this Section, we recall some definitions from graph theory to be used throughout this paper. Let *G* be a simple undirected graph with the *vertex set* *V* = *V*(*G*) and the *edge set* *E* = *E*(*G*). The *order* *n* of *G* is the number of its vertices (*n* = |*V*|). The *size* of *G* is the number of its edges.

**Definition 1**. *The degree (or valency)* deg(*v*) *of a vertex v of a graph G is the number of edges incident to v in G*.

**Definition 2**. *The geodesic distance δ(v, w) of two vertices u and v in graph G is the number of edges of the shortest path in G connecting them*.

**Definition 3**. *The diameter D(G) of a graph G is the maximum of geodesic distances in G*.

By *L*(*G*) we denote the average geodesic distance in graph *G* = (*V*, *E*) [9]:

$$\begin{array}{c} L\left(G\right)=\frac{\sum_{\left\{v,w\right\}\in {\left[V\right]}^{2}}\delta \left(v,w\right)}{n(n-1)} \end{array}$$ (1)

Further, we denote the numerator of the fraction in (1) by Σ(*G*), that is:

$$\begin{array}{c} \Sigma \left(G\right)=\sum_{\left\{v,w\right\}\in {\left[V\right]}^{2}}\delta \left(v,w\right) \end{array}$$ (2)

Thus, (1) can be rewritten as:

$$\begin{array}{c} L\left(G\right)=\frac{\Sigma (G)}{n(n-1)} \end{array}$$ (3)

Further, for every vertex *c* ∈ *V* we denote by Σ(*c*, *G*) the sum of *n* − 1 geodesic distances from *c* to vertices in *V* \ {*c*}. That is:

$$\begin{array}{c} \Sigma \left(c,G\right)=\sum_{u\in V}\delta \left(c,u\right) \end{array}$$ (4)

and using this notation we write

$$\begin{array}{c} \Sigma \left(G\right)=\sum_{u\in V}\Sigma \left(u,G\right) \end{array}$$ (5)

For example, for the *path graph* *P*_2_ on two vertices *u* and *v* connected by an edge we get

$$\begin{array}{c} \Sigma \left(u,P_{2}\right)=\Sigma \left(v,P_{2}\right)=1 \end{array}$$

and

$$\begin{array}{c} \Sigma \left(P_{2}\right)=\Sigma \left(u,P_{2}\right)+\Sigma \left(v,P_{2}\right)=2 \end{array}$$

We repeat the definition of the compactness *C*_*B*_(*G*) of a graph *G* = (*V*, *E*), |*V*| = *n* > 1, as introduced in [2] in a version obtained from [1]:

$$\begin{array}{c} C_{B}\left(G\right)=\frac{K\left(n-1\right)n-(\sum_{\left\{v,w\right\}\in {\left[V\right]}^{2}}\;\delta \left(v,w\right)+K\sum_{G^{\prime }\in Com\left(G\right)}|G^{\prime }\left|(|V|-\right|G^{\prime }\left|)\right)}{\left(K-1)n(n-1\right)} \end{array}$$ (6)

where *K* is “the maximum value an entry in the converted distance matrix [of a graph] can assume” [2, p. 161], *Com*(*G*) is the set of connected components of G and |*G*′| is the order of the graph *G*′ (connected component of the graph *G*). In what follows, we set *K* = *n*. Here, we consider only connected graphs, so we can obviously write the following:

$$\begin{array}{c} C_{B}\left(G\right)=\frac{n}{n-1}-\frac{L(G)}{n-1}. \end{array}$$ (7)

On can easily see that *C*_*B*_(*G*) ≤ 1. Further, since ∀{*v*, *w*} ∈ [*V*]^2^: *D*(*G*) ≥ *δ*(*v*, *w*), we have:

$$\begin{array}{c} 0\leq L(G)\leq D(G). \end{array}$$ (8)

Thus, with (7) and (8) we get for every connected graph *G*:

$$\begin{array}{c} 1\geq C_{B}\left(G\right)=\frac{n}{n-1}-\frac{L(G)}{n-1}=1-\frac{L(G)-1}{n-1}\geq 1-\frac{D(G)-1}{n-1} \end{array}$$ (9)

**Definition 4**. *The path graph P_m_, m* ≥ 2, *is a simple connected undirected graph with two vertices of degree* 1 *(called terminal vertices) and m* − 2 *vertices of degree* 2 *(called internal vertices)*.

The order *n* of *P*_*m*_ is equal to *m* and its diameter *D*(*P*_*m*_) = *m* − 1. The vertices of *P*_*m*_ can be labeled by the consecutive integers {1, 2, …, *m*} in such a way that the terminal vertices are labeled by 1 and *m*, respectively, and for every integer *i*, 1 ≤ *i* ≤ *m* − 1, the consecutive vertices with labels *i* and *i* + 1 are adjacent.

Further we need the following formula the proof of which one can easily get with the straightforward calculation:

$$\begin{array}{c} \Sigma \left(P_{m}\right)=\frac{m(m^{2}-1)}{3} \end{array}$$ (10)

Hence in view of (3) we have

$$\begin{array}{c} L\left(P_{m}\right)=\frac{m(m^{2}-1)}{3m(m-1)}=\frac{m+1}{3} \end{array}$$

and

$$\begin{array}{c} C_{B}\left(P_{m}\right)=\frac{m}{m-1}-\frac{m+1}{3(m-1)}=\frac{2}{3}+\frac{1}{3(m-1)} \end{array}$$ (11)

**Definition 5**. *The Cartesian product G*_1_☐*G*_2_ *of two graphs G*_1_, *G*_2_ *is a graph with vertex set V(G*_1_) × *V(G*_2_ *) such that any two vertices (v, u), (w, z)* ∈ *V(G*_1_☐*G*_2_ *) are adjacent iff v* = *w and u and z are adjacent in G*_2_ *or v and w are adjacent in G*_1_ *and u* = *z*.

**Remark 1**. *If G* = *G*_1_☐*G*_2_ *is the Cartesian product of two graphs G*_1_ *of order n*_1_ *and G*_2_ *of order n*_2_, *then the following properties (referred to below) hold*:

- *G is connected iff both G*_1_ *and G*_2_ *are connected*;
- *the diameter of G is the sum of the diameters of G*_1_ *and G*_2_:
  $$\begin{array}{c} D\left(G_{1}☐G_{2}\right)=D\left(G_{1}\right)+D\left(G_{2}\right) \end{array}$$
- *the order n of G is the product n*_1_ *n*_2_ *of the order n*_1_ *of G*_1_ *and the order n*_2_ *of G*_2_.

**Example 1**. *Let us consider the Cartesian product G(m) of two copies of the path graph P_m_, that is, G(m)* = *P_m_*☐*P_m_ (a so-called square lattice graph whose compactness C_B_ is investigated below). According to Remark 1, G(m) is connected (since P_m_ is connected), its order is m*^2^ *and its diameter D(G(m)) is* 2(*m* − 1).

## Main results

Throughout the present paper we deal with sequences {*G*(*m*)|*m* = 1, 2, …} of connected graphs that satisfy the following “natural” condition

$$\begin{array}{c} \lim_{m\to \infty }n\left(m\right)=\infty \end{array}$$ (12)

where *n* is the order of the graph *G*(*m*).

**Theorem 1**. *Let* {*G(m)*|*m* = 1, 2, …} *be a sequence of simple undirected connected graphs G(m) such that the order n* = *n*(*m*) → ∞ *for m* → ∞. *Assume that the following holds*:

$$\begin{array}{c} lim_{m\to \infty }\frac{D(G(m))}{n}=0, \end{array}$$ (13)

*where D(G(m)) is the diameter of the graph G(m). Then, the compactness C_B_(G(m)) tends to 1 for m* → ∞.

*Proof*. In view of (9) we have

$$\begin{array}{c} 1\geq C_{B}\left(G\left(m\right)\right)\geq 1-\frac{D(G(m))-1}{n-1} \end{array}$$

which implies with our assumptions that

$$\begin{array}{c} \lim_{m\to \infty }C_{B}\left(G\left(m\right)\right)=1 \end{array}$$

**Theorem 2**. *Let {G(m)*|*m* = 1, 2, …} *be a sequence of simple undirected connected graphs G(m) such that the order n* = *n(m)*→∞ *for m* → ∞. *Then, L(G(m))*/*n* →0 *for m* → ∞ (*n* → ∞) *iff D(G(m))*/*n* → 0 *for m* → ∞ (*n* → ∞).

*Proof*. In view of (8), we can easily see that we only need to prove that if *D*(*G*)/*n* ↛ 0 for *m* → ∞ then *L*(*G*)/*n* ↛ 0 for *m* → ∞.

Without loss of generality we assume that

$$\begin{array}{c} \lim_{m\to \infty }D\left(G\right)/n=c\neq 0\;\;\;\left(0<c\leq 1\right) \end{array}$$

Hence, if we take any number *a*, 0 < *a* < *c*, then for all sufficiently large numbers *m* we have

$$\begin{array}{c} D(G)>an \end{array}$$

which implies that there is a geodesic path (subgraph *P*_*k*(*n*)_) in *G* of length *k*(*n*) where *k*(*n*) is the integer part of the number *an*. So we have *an* = *k*(*n*) + *ε*_*n*_ with *ε*_*n*_ (0 ≤ *ε*_*n*_ < 1) being the fractional part of the number *an*. Therefore, in view of Σ(*G*) > Σ(*P*_*k*(*n*)_) and with (10) we have for all sufficiently large *m*

$$\begin{array}{c} L\left(G\right)/n>\Sigma \left(P_{k(n)}\right)/\left(n^{2}\left(n-1\right)\right)=\left(1/3\right)k\left(n\right)\left(k{\left(n\right)}^{2}-1\right)/\left(n^{2}\left(n-1\right)\right). \end{array}$$

Thus, with *k*(*n*) = *an* − *ε*_*n*_ we get

$$\begin{array}{c} L\left(G\right)/n>\frac{\left(an-\varepsilon_{n}\right)\left({\left(an-\varepsilon_{n}\right)}^{2}-1\right)}{3n^{2}\left(n-1\right)}=\frac{\left(a-\varepsilon_{n}/n\right)\left({\left(a-\varepsilon_{n}/n\right)}^{2}-1/n^{2}\right)}{3(1-1/n)} \end{array}$$

which implies in view of lim_*m*→∞_ *n* = ∞ that for all sufficiently large numbers *m* we have

$$\begin{array}{c} \frac{L(G)}{n}\geq a^{3}/4>0. \end{array}$$

Hence, *L*(*G*)/*n* ↛ 0.

From these two theorems obviously follows:

**Corollary 1**. *For any sequence of simple undirected connected graphs G(m) for which the order n* = *n(m) of G(m) tends to* ∞ *whenever m* → ∞, *C_B_(G(m))* → 1 *for m* → ∞ *iff for m* → ∞ *L(G(m))*/*n* → 0 (*D(G(m))*/*n* → 0).

And what about the case of disconnected graphs? It turns out that Corollary 1 can be easily generalized with some consideration for the case of disconnected graph classes. That is, the following statement holds:

**Theorem 3**. *Let* {*G*(*m*)|*m* = 1, 2, …} *be a sequence of simple undirected not necessarily connected graphs G(m) such that the order n* = *n*(*m*) → ∞ *for m* → ∞. *Then, the compactness C*_*B*_ *of G(m) tends to* 1 *iff both of the following equalities hold*:

1. *L*(*G*(*m*))/*n* →0 (*or D*(*G*(*m*))/*n* → 0) *for m* → ∞
2. lim_*m*→∞_ *n*_1_/*n* = 1 *(equivalently*, lim_*m*→∞_(*n* − *n*_1_)/*n* = 0), *where n*_1_ *is the order of the largest connected component of G(m)*.

These results give an answer to the question in which case *C*_*B*_(*G*(*m*)) tends to 1 for *m* → ∞ (*n* → ∞).

## Some simple applications

In this section, we consider four simple classes of undirected connected graphs and examine their compactness *C*_*B*_ in the limit of their order (i.e., *n* → ∞). Sometimes, *C*_*B*_ is easily estimated as in the case of complete graphs. In most cases, however, it is difficult to calculate the exact value of *C*_*B*_ or to give a good estimation of it. Here, we refer to Corollary 1 in order to do this.

The examples of graphs considered here have the following properties. Their diameter *D*(*G*) is either constant or grows slower than its order *n* in such a way that *D*(*G*)/*n* tends to 0 whenever *n* tends to ∞.

### Complete graphs

A *complete graph* *K*_*n*_ of order *n* is a simple undirected graph with *n* vertices such that each pair of distinct vertices is connected by a unique edge. That is, the average geodesic distance *L*(*K*_*n*_) and the diameter *D*(*K*_*n*_) both are equal to 1. Using (7), we get the exact value of compactness *C*_*B*_(*K*_*n*_) of *K*_*n*_:

$$\begin{array}{c} C_{B}\left(K_{n}\right)=\frac{n}{n-1}-\frac{L(G)}{n-1}=\frac{n}{n-1}-\frac{1}{n-1}=1 \end{array}$$

It is worth noting that the complete graph *K*_*n*_ is the only graph for which *C*_*B*_(*K*_*n*_) equals 1. This trivially results in the following equality:

$$\begin{array}{c} \lim_{n\to \infty }C_{B}\left(K_{n}\right)=1 \end{array}$$

The same result is obtained by directly applying Corollary 1.

### Star graphs

A *star graph* *S*_*m*_ on *m* vertices (*m* > 2) is a simple undirected connected graph in which one vertex called *central* vertex has degree *m* − 1 and another *m* − 1 vertices have degree 1.

Consider a sequence {*S*_*m*_|*m* = 1, 2, …} of connected star graphs of order *n* = *m* where diameter *D*(*S*_*m*_) is obviously equal to 2 ∀*m* ≥ 3. By using Corollary 1 we immediately obtain (seemingly counter-intuitively to what we expect should be measured by compactness):

$$\begin{array}{c} \lim_{m\to \infty }C_{B}\left(S_{m}\right)=1 \end{array}$$

It is worth noting that for *S*_*m*_ (*m* > 2) it is easy to calculate the value of *L*(*S*_*m*_) and of *C*_*B*_(*S*_*m*_). Indeed, we have

$$\begin{array}{c} \Sigma \left(S_{m}\right)=2{\left(m-1\right)}^{2} \end{array}$$

where Σ(*G*) is defined by (2). Next, using (3), *L*(*S*_*m*_) can be computed as follows:

$$\begin{array}{c} L\left(S_{m}\right)=2{\left(m-1\right)}^{2}/\left(m\left(m-1\right)\right)=2\left(m-1\right)/m \end{array}$$

So with (7) we clearly have

$$\begin{array}{c} C_{B}\left(S_{m}\right)=\frac{m}{m-1}-\frac{2}{m} \end{array}$$

Hence, it follows that *C*_*B*_(*S*_*m*_) → 1 as *m* → ∞. Thus, we get the same result as in the case of complete connected graphs by calculating *C*_*B*_(*S*_*m*_) without Corollary 1.

### Lattice graphs

We consider a simple undirected graph *G*(*m*) whose vertices can be associated with the points in the plane with the integer *x* and *y* coordinates being both in the range 1, 2, …*m*. Two vertices are connected by an edge if and only if the distance between them is equal to 1. Such a graph is called a *lattice graph* or a square grid graph and can be viewed as the Cartesian product of two copies of the path graph *P*_*m*_, that is, *G*(*m*) = *P*_*m*_☐*P*_*m*_ (see Example 1). So, we only repeat that *G*(*m*) is connected, has the order *n* = *m*^2^ and the diameter *D*(*G*(*m*)) = 2(*m* − 1).

Let us consider a sequence {*G*(*m*)|*m* = 1, 2, …} of lattice graphs. What is the limit value of *C*_*B*_(*G*(*m*))? With *n* = *m*^2^ and *D*(*G*(*m*)) = 2(*m* − 1) we have

$$\begin{array}{c} \lim_{m\to \infty }n=\infty \end{array}$$

and

$$\begin{array}{c} \lim_{m\to \infty }\frac{D(G(m))}{n}=\lim_{m\to \infty }\frac{2(m-1)}{m^{2}}=0 \end{array}$$

Hence, with Corollary 1 we immediately have lim_*m*→∞_ *C*_*B*_(*G*(*m*)) = 1.

### Hypercube graphs

A *hypercube graph* *Q*_*m*_ is a simple undirected connected graph on 2^*m*^ vertices labeled by the numbers 0, 1, …, 2^*m*^ − 1. Two vertices are connected by an edge if and only if the binary representations of their labels differ exactly in one position. *Q*_*m*_ can also be defined as the Cartesian product of *m* copies of the path graph *P*_2_:

$$\begin{array}{c} Q_{m}=\underset{m}{\underset{︸}{P_{2}☐P_{2}☐\ldots ☐P_{2}}} \end{array}$$

In view of Remark 1 of Section, we see that *Q*_*m*_ is connected (because *P*_2_ is connected), its diameter *D*(*Q*_*m*_) equals *m* and its order *n* is 2^*m*^. We easily see that the fraction *D*(*Q*_*m*_)/*n* = *m*/2^*m*^ tends to zero for *m* → ∞, so with Corollary 1 we obtain:

$$\begin{array}{c} \lim_{m\to \infty }C_{B}\left(Q_{m}\right)=1. \end{array}$$

## Concluding remarks

We confined us here to providing only four simple examples of the graph classes, for which our tool can be easily applied. Actually, we have found more than 30 well-known graphs classes for which our tool is applicable. So, the compactness of these graphs tends to 1 whenever their order tends to ∞.

First, among these graph classes there are those whose diameter does not depend on the order *n*. These are *book graphs*, *complete r-partite graphs*, *crown graphs*, *Hadamard graphs*, *Keller graphs*, *lating square graphs*, *Paley graphs*, *strongly regular graphs*, *Turán graphs*, *wheel graphs*, *windmill graphs* and some others.

Next, we found some graph classes for each of which the estimation of its diameter as a function of the order *n* allows the application of our tool. Among those graph classes are the following: *de Bruijn graphs*, *cube-connected cycles*, *Fibonacci cube graphs*, *folded cube graphs*, *Hamming graphs*, *Johnson graphs*, *king’s graphs*, *Kneser graphs*, *knight’s graphs*, *perfect undirected binary trees*, *self-complementary graphs*, *Ramanujan graphs* and others.

Further, we have seen in Section that the complete graph *K*_*m*_ has the largest possible value of compactness which is 1. So we can say that the graph *K*_*m*_ is the most compact graph among all the graphs of the same order *m*. If we try now to get the limit value of compactness of the path graph *P*_*m*_ using our tool, we see that this is not possible because *D*(*P*_*m*_)/*m* ↛ 0 for *m* → ∞. Indeed,

$$\begin{array}{c} \lim_{m\to \infty }\frac{D(P_{m})}{m}=\lim_{m\to \infty }\frac{m-1}{m}=1 \end{array}$$

but with (11) we easily get lim_*m*→∞_ *C*_*B*_(*P*(*m*)) = 2/3. We can prove (to appear) that the path graph *P*_*m*_ is the least compact among all the simple connected undirected graphs of the same order *m*. That is, for each such graph *G* of order *m* the following holds:

$$\begin{array}{c} \frac{2}{3}<C_{B}\left(P_{m}\right)\leq C_{B}\left(G\right))\leq C_{B}\left(K_{m}\right)=1. \end{array}$$

This finding defines the range of possible values of compactness for connected graphs. Hence, the limit value of compactness for any sequence of simple connected undirected graphs lies within the interval [2/3; 1]. Moreover, we can prove (to appear) that for any number *α* in the interval [2/3; 1] a graph family can be constructed for which the limit value is exactly *α*.

It is worth noting that in the case of not necessarily connected graphs the value of compactness lies within the interval [0, 1]. Our future work will consider, amongst others, an extended set of graph classes and the study of a range of invariants including weighted and unweighted ones.
